# Co_2_MnO_4_/Ce_0.8_Tb_0.2_O_2−δ_ Dual-Phase Membrane Material with High CO_2_ Stability and Enhanced Oxygen Transport for Oxycombustion Processes

**DOI:** 10.1021/acsaem.3c02606

**Published:** 2023-12-18

**Authors:** Marwan Laqdiem, Julio Garcia-Fayos, Alfonso J. Carrillo, Laura Almar, María Balaguer, María Fabuel, José M. Serra

**Affiliations:** Instituto de Tecnología Química (Universitat Politècnica de València − Consejo Superior de Investigaciones Científicas), Av. Los Naranjos s/n, E-46022 Valencia, Spain

**Keywords:** oxygen permeation, dual-phase, CO_2_, surface reactions, oxycombustion

## Abstract

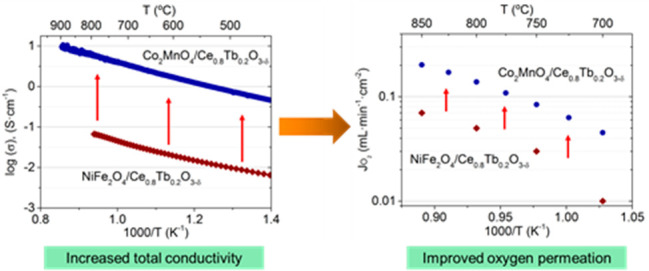

Oxygen transport membranes (OTMs) are a promising oxygen production technology with high energy efficiency due to the potential for thermal integration. However, conventional perovskite materials of OTMs are unstable in CO_2_ atmospheres, which limits their applicability in oxycombustion processes. On the other hand, some dual-phase membranes are stable in CO_2_ and SO_2_ without permanent degradation. However, oxygen permeation is still insufficient; therefore, intensive research focuses on boosting oxygen permeation. Here, we present a novel dual-phase membrane composed of an ion-conducting fluorite phase (Ce_0.8_Tb_0.2_O_2−δ_, CTO) and an electronic-conducting spinel phase (Co_2_MnO_4_, CMO). CMO spinel exhibits high electronic conductivity (60 S·cm^–1^ at 800 °C) compared to other spinels used in dual-phase membranes, i.e., 230 times higher than that of NiFe_2_O_4_ (NFO). This higher conductivity ameliorates gas–solid surface exchange and bulk diffusion mechanisms. By activating the bulk membrane with a CMO/CTO porous catalytic layer, it was possible to achieve an oxygen flux of 0.25 mL·min^–1^·cm^–2^ for the 40CMO/60CTO (%_vol_), 680 μm-thick membrane at 850 °C even under CO_2_-rich environments. This dual-phase membrane shows excellent potential as an oxygen transport membrane or oxygen electrode under high CO_2_ and oxycombustion operation.

## 1 Introduction

Oxygen transport membranes (OTMs) are considered one of the technologies contributing to mitigating climate change due to their application in carbon capture and storage (CCS) strategies, leading to avoiding greenhouse-effect gas emissions.^1,2^ The production of pure oxygen opens the gate to changing traditional combustion to oxycombustion processes, which are more efficient and more accessible to capture CO_2_ from the exhaust.^3,4^ Cryogenic air distillation is the conventional technology for pure O_2_ production.^2^ This technology is mature and efficient for large amounts of oxygen production, 30,000 Nm^3^·h^–1^, with a 99% purity.^5^ However, its efficiency decreases for lower oxygen production capacity because it is produced using extensive equipment and critical conditions like high vacuum and very low temperature.^2,5^

OTMs can be adapted to existing processes and have a more comprehensive operational range than other technologies for oxygen production.^6−8^ OTMs could be adapted to combustion processes or used as catalytic membrane reactors (CMRs).^9^ In both processes, the OTMs should be operational and stable in contact with CO_2_ atmospheres; e.g., the permeate sweep gas used in this process is part of the CO_2_ produced in the oxycombustion.^4^ OTMs’ traditional materials are based on oxides with perovskite crystal structures (ABO_3_) like Ba_0.5_Sr_0.5_Co_0.8_Fe_0.2_O_3−δ_ or La_0.6_Sr_0.4_Co_0.8_Fe_0.2_O_3−δ_.^10−14^ However, these materials are unstable in CO_2_ atmospheres at high temperatures (700–1000 °C) since the alkaline earth reacts with the CO_2_, producing the corresponding carbonates or remaining adsorbed, blocking the active sites and decreasing oxygen permeation.^15^

Solving the stability issue is still a challenge. Recently, a combination of materials stable in CO_2_ (with electronic and/or ionic conductivity) as dual-phase membranes have been proposed as potential candidates.^16−20^ Usually, the ionic phase is based on doped zirconia or doped ceria materials. These materials have fluorite crystal structure and are stable in CO_2_ atmospheres. Also, the ionic conductivity of these oxides depends on the specific lattice composition. The most used doped zirconia materials are yttria-stabilized zirconia (YSZ) and scandia-doped zirconia (ScSZ).^21−23^ In the case of doped ceria, among the available dopants used for increasing the ionic conductivity, the most common are gadolinium-doped (CGO) and samarium-doped ceria (CSO).^24,25^ Ceria has also been doped with praseodymium or terbium, increasing its mixed ionic electronic conductivity.^26,27^ On the contrary, a broader range of possible materials is available for the electronic phase.^5^ In general, they could be classified by their crystal structure. The three general crystal structures for the electronic or mixed ionic electronic conductor (MIEC) phases are simple perovskite, spinel, and Ruddlesden–Popper.^20,28−31^ Among these, the most used materials present a perovskite crystal structure. In this context, these oxide structures can be MIEC or pure electronic semiconductors, but most of them comprise alkaline earth in the material and hence involve stability challenges under CO_2_ atmospheres at mid-long times of operation.^17,19,28^ Spinels are the second material class most used for the electronic phase in dual-phase membranes. Most of these materials are stable in CO_2_, but many, especially those containing Co, Ni, or Cu, decompose in reducing atmospheres (*p*O_2_ < 10^–15^ bar).^31−34^ Ruddlesden–Popper phases, typically comprising Co, Ni, Fe, or Cu cations, are generally MIEC materials with a high oxygen transport capacity. Still, they usually are not entirely stable in CO_2_ and present instabilities under different atmospheres (*p*O_2_ between 10^–1^ and 10^–5^ bar) on both membrane sides.^20,35,36^

The dual-phase material NiFe_2_O_4_/Ce_0.8_Tb_0.2_O_2−δ_ (NFO/CTO) combines the spinel and fluorite phases. This composite material exhibits high stability in CO_2_ atmospheres and can even be stable in the presence of low amounts of SO_2_ without permanent degradation.^31−33^ However, this material shows limited permeation rates compared to other dual-phase materials.^5^

In this sense, this study’s primary goal is to improve the permeation for this type of dual-phase OTMs. For that purpose, 20% terbia-doped ceria (Ce_0.8_Tb_0.2_O_2−δ_, CTO) was chosen as the ionic conductor material, and Co_2_MnO_4_ (CMO) replaced the NiFe_2_O_4_ phase (NFO)^37^ to study the effect of changing the electronic phase in comparison to previous works.^16,32,33,40^ The rationale behind the choice of CMO was its high electronic conductivity compared to NFO. Namely, the electric conductivity for CMO and NFO is 60 and 0.26 S·cm^–1^ at 800 °C, respectively.^37^ In addition, this material is of some interest as a cathode for solid oxide fuel cells (SOFCs) because it is commonly used as a current collector in SOFC stacks.^38,39^ Improving the electrical conductivity could allow for a lower amount of the electronic phase, thus increasing the amount of the ionic conductivity phase while remaining stable under CO_2_. This would result in higher ambipolar transport, thus boosting oxygen permeation. The ratio of the dual-phase (Co_2_MnO_4_/Ce_0.8_Tb_0.2_O_2−δ_) material was fixed to 40% vol. for the electronic phase and 60% vol. for the ionic phase. This study introduces this dual-phase material as a promising candidate for the use of OTMs in oxycombustion processes, improving oxygen permeation in CO_2_ gas environments.

## 2 Experimental Section

### 2.1 Materials Synthesis and Sample Preparation

Co_2_MnO_4_ (CMO) and Ce_0.8_Tb_0.2_O_2−δ_ (CTO) ceramic powders were synthesized to fabricate membranes, porous catalytic layers for oxygen permeation studies, bars, and porous electrodes (as catalytic layers) for electrochemical characterization studies. The one-pot Pechini method was used to prepare the dual-phase materials. This method provides better homogeneity and smaller particle size than the solid-state reaction.^41^ Stoichiometric amounts of Ce(NO_3_)_3_·6H_2_O, Tb(NO_3_)_3_·6H_2_O, and Co(NO_3_)_2_·6H_2_O provided by Sigma-Aldrich and Mn(NO_3_)_2_·4H_2_O from Alfa Aesar were mixed in a homogeneous aqueous solution. The ratio for each phase was 40% vol. of CMO and 60% vol. of CTO. Afterward, the nitrates were dissolved, citric acid (Sigma-Aldrich) was added as a chelating agent to prevent partial segregation of metal components, and ethylene glycol was added to polymerize with the chelating agent, producing an organometallic polymer (in a molar ratio of 1:2:4, respectively). This complexation is followed by dehydration at 220 °C and thermal decomposition at 850 °C for 5 h to form the desired spinel and fluorite phases. Dense membrane disks of 26 mm diameter and rectangular bars with 6.9 × 1.7 × 4.2 mm^3^ dimensions (for conductivity measurements) were pressed and then calcinated at 1200 °C for 5 h. In addition, for electrochemical impedance spectroscopy (EIS) studies, uniaxially pressed Ce_0.8_Gd_0.2_O_2−δ_ (CGO82) electrolyte disks of 26 mm diameter were sintered at 1450 °C for 10 h. The sintered electrolytes and membranes were reduced to a diameter of 15 mm and a thickness of 0.65 mm by polishing with sandpaper. Porous catalytic layers (∼30 μm and 9 mm in diameter) of the CMO/CTO composite ink were screen-printed on the dense membranes or electrolytes. Screen-printing inks were made by mixing a 1:1 final weight ratio of the considered ceramic powders and an ethyl cellulose (6% wt.) solution in terpineol in a three-roll mill. The screen-printed porous layer was dried and sintered at 950 °C in air for 2 h. In this study, the dense oxygen transport membrane had a thickness of 680 μm and 30–35 μm of porous catalytic layer on both sides.

### 2.2 Characterization of Materials and Membranes

Crystalline phases of produced samples were identified using a PANalytical Cubix fast diffractometer, Cu Kα_1,2_ radiation (λ_1_ = 1.5406 Å), and an X’Celerator detector in Bragg–Brentano geometry. XRD patterns recorded in the 2θ range from 20° to 80° were analyzed using X’Pert Highscore Plus software. Cross-section analysis of the sintered materials before and after the permeation and electrochemical tests was conducted by scanning electron microscopy (SEM) using a GeminiSEM 500 from Zeiss. A backscattered electron detector (BSD) was used to provide images with compositional contrast that differentiates grains and elements distribution. Furthermore, energy-dispersive X-ray spectroscopy (EDS) was used to analyze cross-section images of the post-mortem dual-phase membranes. The EDS used was a ZEISS Ultra55 field emission SEM.

**Figure 1:**
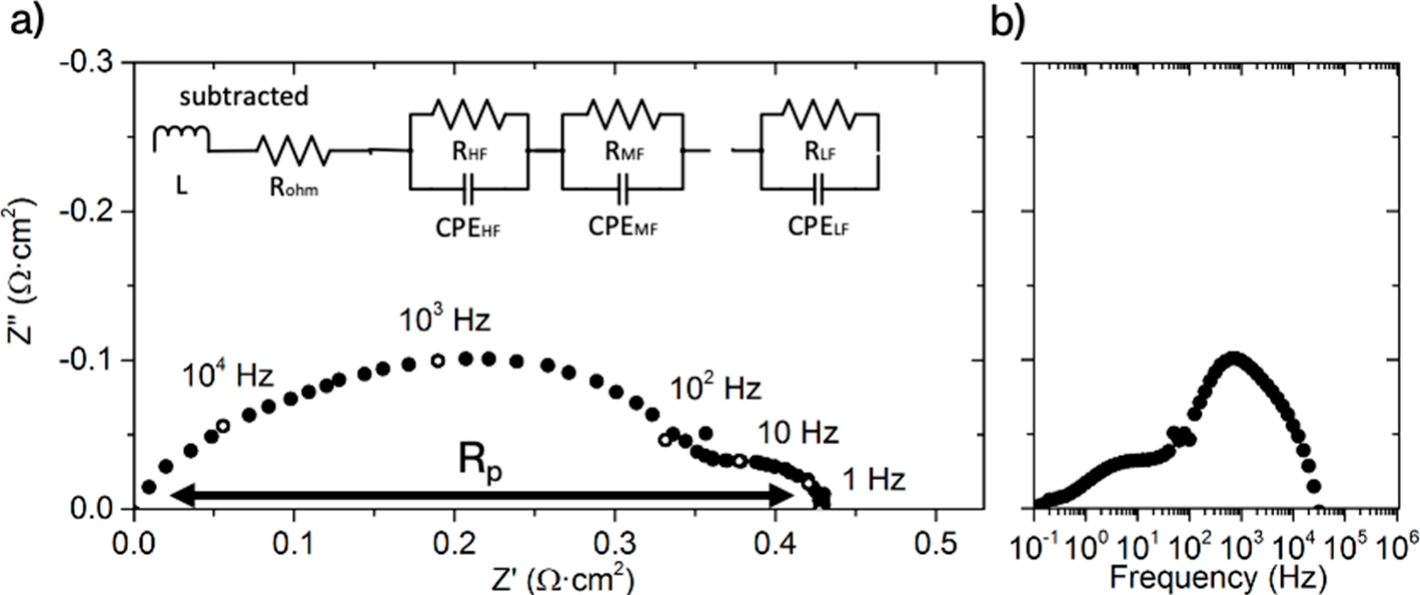
Impedance spectra in air at different temperatures (*T* = 850–750 °C) for symmetrical cells with 40CMO/60CTO electrodes. (a) Nyquist and (b) Bode plots (ohmic losses were subtracted for clarity reasons).

Electrochemical impedance spectroscopy (EIS) was carried out to characterize the CMO/CTO porous catalytic layers and study the surface exchange reaction. The lab-scale reactor consisted of a cylindrical quartz reactor, and the samples were placed between 2 meshes of Au used as current collectors. Two sets of experiments were conducted, first, analyzing the influence of temperatures from 850 to 750 °C in air and, second, the effect of varying the oxygen partial pressures from 10% air in Ar to 100% air both at 850 °C. The total flow used was 200 mL·min^–1^. In this case, the chambers have the same mixture flow. EIS was performed with a Solartron 1470/1455 FRA instrument used with a 0 V DC and 20 mV AC amplitude signal. The ZView software was employed using the equivalent circuit shown in Figure 1 to analyze and fit the impedance spectra. The inductance and the ohmic resistance are subtracted, and the contribution at low frequencies was used only when visible. The impedance spectra were fitted to the equivalent circuit *R*_p_ = *R*_HF_ + *R*_MF_ + *R*_LF_. High frequencies (RHF) are higher than 1000 Hz, mid frequencies (RMF) are between 1000 and 10 Hz, and low frequencies (*R*_LF_) are lower than 10 Hz.

Permeation tests were conducted in a lab-scale reactor consisting of a cylindrical quartz reactor with two chambers separated by the sealed sample membrane, in which synthetic air (21% vol. O_2_) or pure O_2_ was fed into the oxygen-rich chamber (100–150 mL·min^–1^). Ar and CO_2_ were used as sweep gas on the permeate side chamber (150 mL·min^–1^) in a 4-end mode configuration. Both streams were fed at an atmospheric pressure. Inlet gases were preheated to ensure isothermal operation and prevent the possibility of thermal shock in contact with the membrane surface. This is particularly important when high gas flow rates are employed. All streams were individually mass-flow controlled. The temperature was measured with a K-type thermocouple close to the membrane. Membrane gas leak-free conditions were achieved by using sealants based on Ag alloy rings. The sealing temperature was between 850 and 860 °C. The permeate was analyzed at a steady state by online gas chromatography using a micro-GC Varian CP-4900 equipped with Molsieve5A, Pora-Plot-Q glass capillary, and CP-Sil modules. Membrane gas leak-free conditions were ensured by continuously monitoring the N_2_ concentration in the product gas stream.

## 3 Results and Discussion

### 3.1 Sample Characterization and Total Conductivity

**Figure 2:**
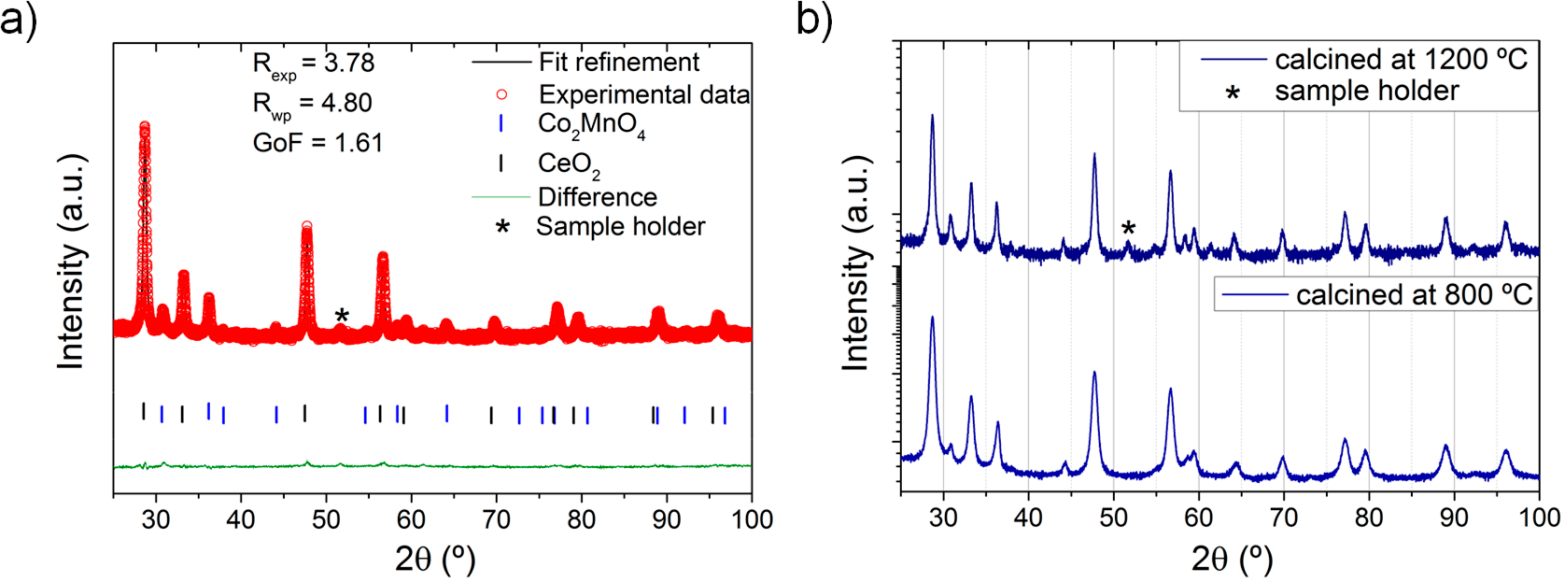
(a) Rietveld refinement for CMO/CTO calcined at 1200 °C for 5 h. The PDF card for CMO was 01-084-0482, and that for CTO was 01-083-5824. (b) X-ray diffraction for CMO/CTO after being calcined at 800 °C (bottom) and 1200 °C (top).

The crystal structure and phase purity of the synthesized CMO/CTO samples observed by X-ray diffraction (XRD) are shown in Figure 2. The Rietveld refinement of the membrane sample sintered at 1200 °C (Figure 2a) reveals the composition: 45.7% vol. of CMO and 54.3% vol. of CTO for the dual-phase material. Figure 2b shows the XRD pattern for the dual-phase material calcined at 800 °C for 5 h (at the bottom) and for the rectangular bar at 1200 °C for 5 h (at the top). Here, the X-ray diffraction patterns for both phases revealed the absence of detectable impurities. The diffraction peak position was maintained, indicating that the dual-phase material did not undergo any compositional change upon annealing at higher temperatures. The sharper peaks observed for the sample calcined at 1200 °C illustrate the expected increase in crystallinity, maintaining the principal peaks for the CTO and CMO in 28.7° and 36.2°, respectively. The compatibility of both phases—even reached via one-pot synthesis—is remarkable, yet other related fluorite–spinel composites, such as Ce_0.8_Gd_0.2_O_1.9_–FeCo_2_O_4_,^42^ lead to secondary phases, e.g., perovskites, along the grain boundaries.

To assess the density of the membranes, cross-section images were taken by performing scanning electron microscopy (SEM) (Supporting Information, Figure S1a). SEM analysis confirms that the membrane is highly dense, although some occlusive porosity can be observed. Furthermore, the backscattered electron detector (BSD) was used to see both phases with good percolative paths (Figure S1b). Therefore, ambipolar diffusion through the dual-phase membrane could occur without percolative impediment,^43^ thus increasing oxygen diffusion.

One of the main objectives of this study was to influence the oxygen permeation of the dual-phase spinel/fluorite materials by tailoring the electronic conductivity of the composite, i.e., using CMO as a better electronic conductor than NFO. The electronic conductivity difference for these two materials is ascribed to the conduction through different carriers. According to Šutka and Gross, NiFe_2_O_4_ crystallizes in an inverse spinel structure in which Ni^2+^ cations are located in the octahedral sites of the spinel and presents n-type conductivity, which is based on hole (h+) hopping between Ni^2+^ and Ni^3+^ in octahedral sites (Ni^2+^ + h^+^ ↔ Ni^3+^).^44^ However, NFO can change to p-type depending on the fabrication method, especially when the fabrication method leads to higher Ni^3+^/Ni^2+^ ratios.^45^ In that case, the conductivity mechanism will be based on electron hopping between Fe^3+^ + e– ↔ Fe^2+^. On the other hand, Co_2_MnO_4_ has p-type conductivity.^46^ In this spinel, both Co^3+^ and Mn^3+^ cations are located in the octahedral sites, and conductivity is based on electron hopping on these species, especially between Mn^3+^ and Mn^4+^. It is possible that both the differences between the conductivity mechanism and the electronic configuration gave rise to the huge difference in electrical conductivity, which could also be associated with favorable (less energy demanding) redox requirements of Mn oxidation/reduction transitions if compared to Fe ones. Increasing the electric conductivity is expected to have an impact on oxygen permeation, following the Wagner equation
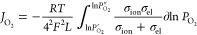
1where *R* is the gas constant, *T* is the membrane temperature, *F* is the Faraday constant, and *L* is the membrane thickness; ln *P*_O_2__^″^ and ln *P*_O_2__^′^ are the oxygen partial pressures for the sweep and the feed chamber;  is the ambipolar conductivity (σ_amb_), formed by the product of ionic and electronic conductivity and divided by the total conductivity.^47^

**Figure 3:**
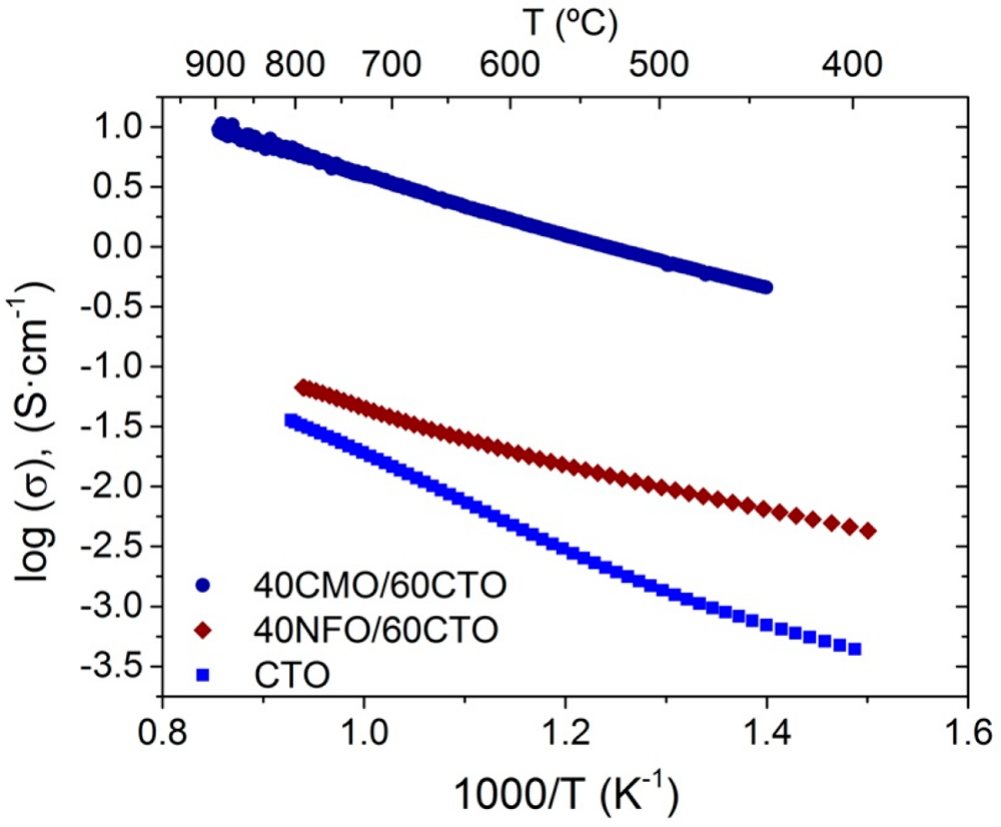
Total conductivity in air at different temperatures for the dual-phase materials 40CMO/60CTO (this study), 40NFO/60CTO, and CTO.^16^

In this study, the total conductivity of the CMO/CTO material was measured and is shown in Figure 3. Also, it was compared with the CTO and other dual-phase material (40NFO/60CTO), a very stable material in CO_2_.^16,32^ Remarkably, the total conductivity of the CMO/CTO material is more than 1 order of magnitude higher than that of the NFO/CTO composite, 0.77 and 0.07 S·cm^–1^, respectively, at 800 °C in air.^16^ The CTO has a total conductivity of around 0.034 S·cm^–1^ at 800 °C in air.

### 3.2 Electrochemical Studies

**Figure 4:**
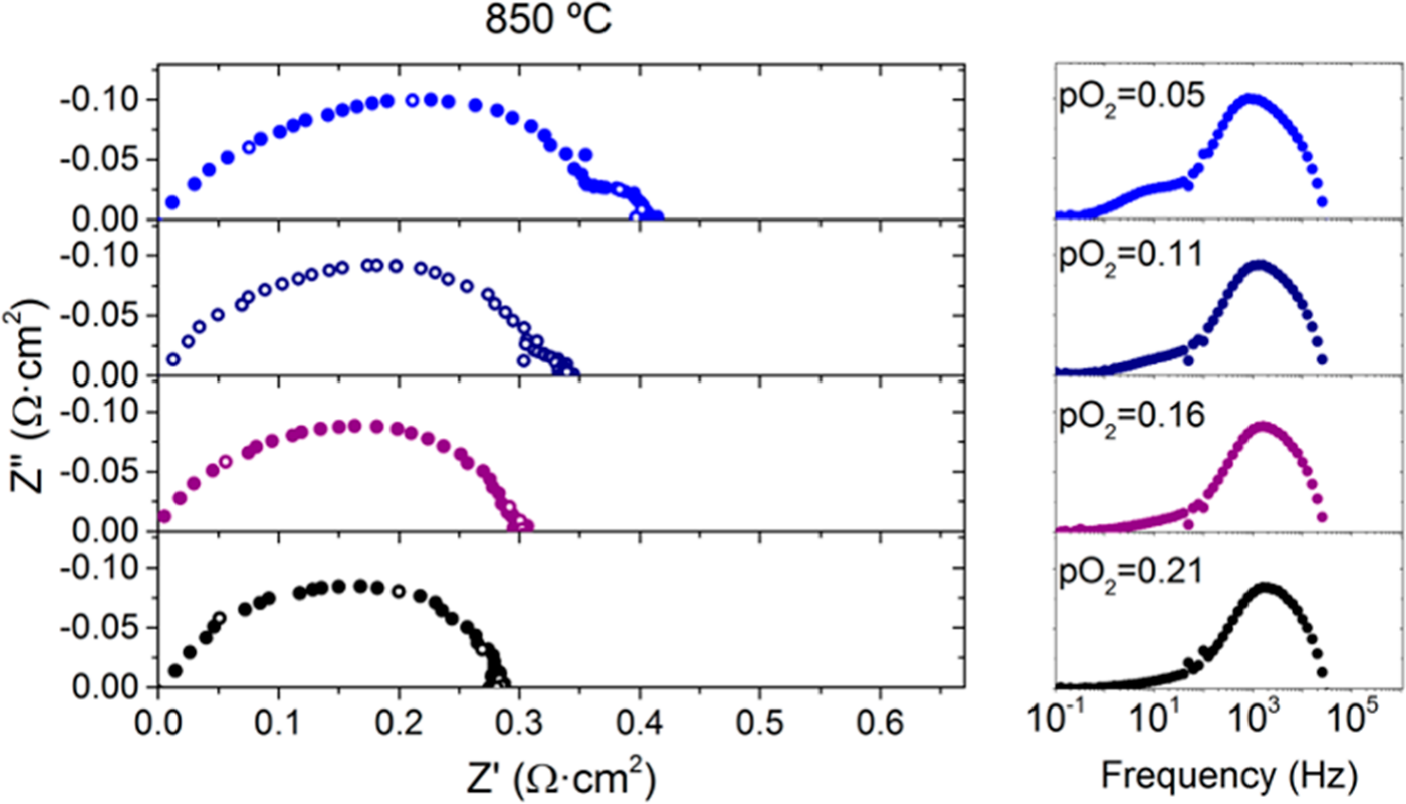
Impedance spectra for symmetrical cells with 40CMO/60CTO electrodes (Nyquist and Bode diagram) for dual-phase material 40CMO/60CTO as the electrode at 850 °C at different *p*O_2_ values (*p*O_2_ = 0.21–0.05 bar) (ohmic losses were subtracted for clarity reasons).

The electrochemical properties of this dual-phase material were characterized by EIS. The dual-phase material was deposited as a porous electrode on a disk-shaped CGO electrolyte. This study entails two parts: (i) evaluating the influence of *p*O_2_ at 850 °C and (ii) the effect of temperature, studied under different atmospheres, namely, air, 5% O_2_/95% Ar, and 5% O_2_/95% CO_2_. These studies aim to discern the limiting steps on surface reactions for this dual phase as oxygen-exchange activation layers under OTM operational conditions. A previous study based on NFO/CTO dual-phase materials shows how polarization resistances obtained by EIS studies directly correlate with oxygen permeation.^48^ To investigate the influence of different oxygen concentrations in the surface exchange reactions, the composite material was studied by EIS for different *p*O_2_ values at 850 °C, represented in Figure 4 as Nyquist (a) and Bode (b) diagrams. The resistance for the different contributions follows a power law with the *p*O_2_ as shown in eq 2, where the exponential number is characteristic of the controlling step in the surface exchange reaction mechanism: eq 3 to eq 6.^49^
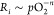
2
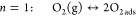
3
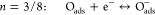
4
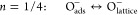
5
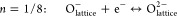
6

**Figure 5:**
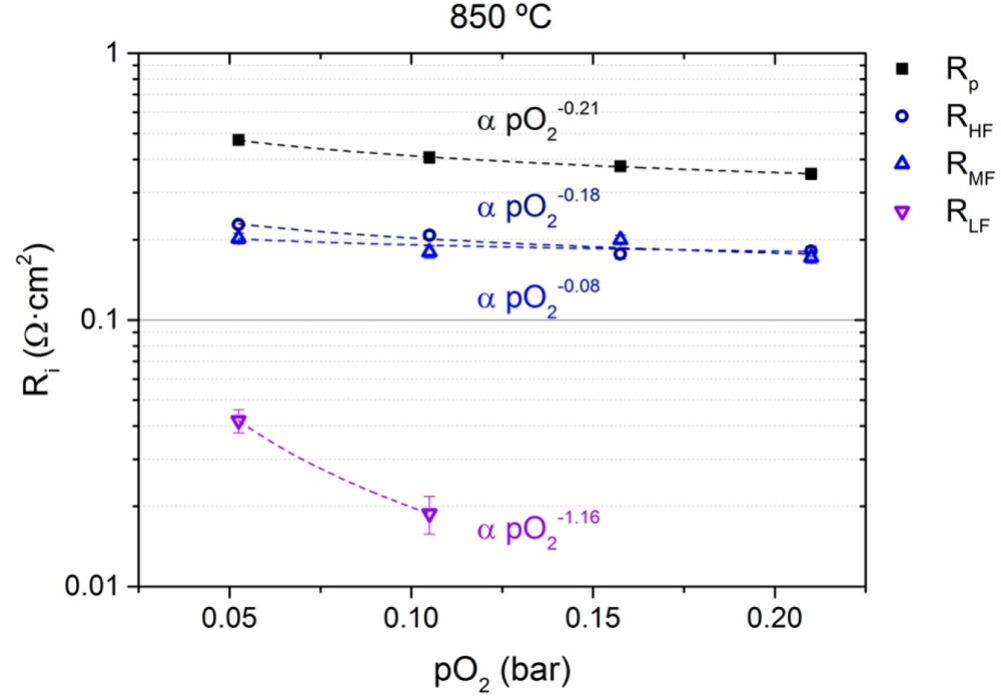
Polarization resistance (*R*_p_) and different fitted resistances (*R*_HF_, *R*_MF_, and *R*_LF_) measured at 850 °C at different *p*O_2_ values (from 0.21 to 0.05 bar) for symmetrical cells with 40CMO/60CTO electrodes.

Decreasing the oxygen concentration does not reveal a significant increase in the initial surface contributions. However, at *p*O_2_ values of 0.11 and 0.05 bar, another contribution appears at low frequencies (lower than 10 Hz). The impedance spectra were fitted to the equivalent circuit *R*_p_ = *R*_MF_ + *R*_HF_ + *R*_LF_, Table S1. The results for *R*_p_ and each contribution resistance are shown in Figure 5 for the *p*O_2_ range of 0.21–0.05 bar at 850 °C.

Here, the resistance at low frequencies (*R*_LF_) can be associated with the oxygen gas diffusion and adsorption (exponential factor 1.16, close to 1 as in eq 2).^48^ The *R*_MF_ contribution at mid frequencies and the *R*_HF_ contribution at high frequencies are very similar. Both can be associated with oxygen-ion adsorption through both phases (exponential factor 0.08 for *R*_MF_ and 0.18 for *R*_HF_, near 1/8 as in eq 6).^50,51^

**Figure 6:**
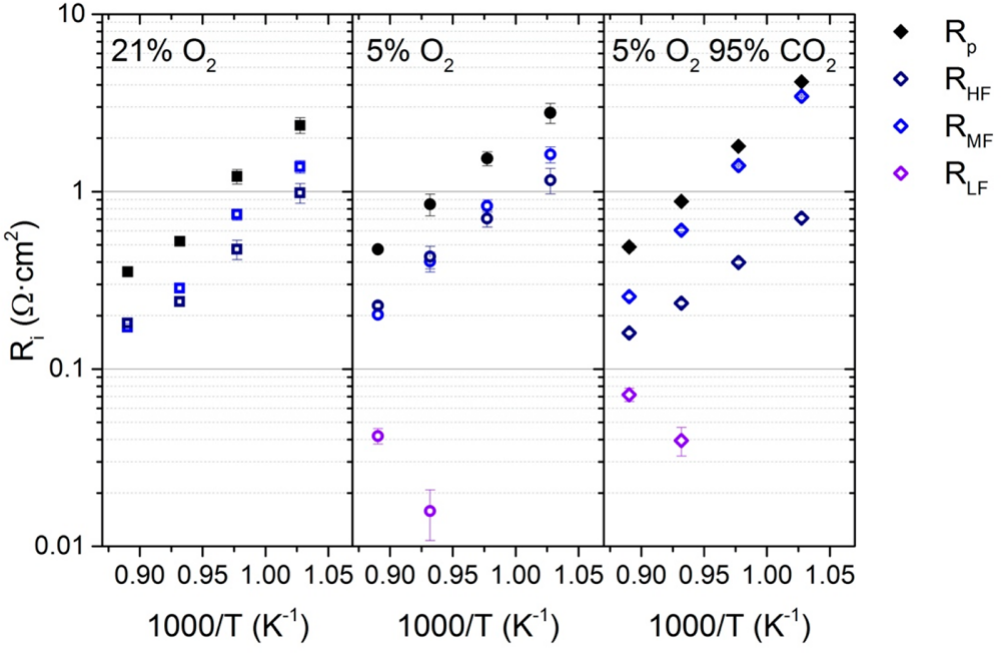
Polarization resistance (*R*_p_) and different fitted resistances (*R*_HF_, *R*_MF_, and *R*_LF_) measured at different environments (air, 5% O_2_ in Ar, and 5% O_2_ in CO_2_) from 850 to 750 °C for symmetrical cells with 40CMO/60CTO electrodes.

Figure S2 collects the Nyquist and Bode diagrams for the CMO/CTO porous catalytic layer from 850 to 750 °C in air at 5% O_2_/95% Ar and 5% O_2_/95% CO_2_. It can be observed that the lower the temperature, the higher the polarization resistance (*R*_p_), following monotonic Arrhenius behavior. At 850 °C, the polarization resistance (*R*_p_) for the three environments is very similar, but at 700 °C, the polarization resistance for CO_2_ environments differs from the others. These EIS data were fitted to the equivalent electrical circuit, *R*_p_ = *R*_MF_ + *R*_HF_ + *R*_LF_ (Tables S2, S3, and S4), as shown in in Figure 6. It is also interesting to mention how the highest contributions shift to lower frequencies when the temperature decreases, as shown in Figure S3 (Supporting Information). At 700 °C in CO_2_ environments, the low frequency and mid frequency contributions are combined, with a maximum frequency of 23 Hz.

Here, for the dual-phase catalytic layer in air, the *R*_p_ increases from 0.35 Ω·cm^2^ at 850 °C to 2.36 Ω·cm^2^ at 750 °C. This performance is similar at 5% O_2_ in Ar, obtaining 0.47 Ω·cm^2^ at 850 °C and 2.78 Ω·cm^2^ at 750 °C. On the contrary, when the environment changes to 5% O_2_ in CO_2_, the polarization resistance is similar at the other environments at 850 °C, but it increases 1 order of magnitude at 750 °C, 0.49 Ω·cm^2^, and 4.15 Ω·cm^2^, respectively. In this study, both principal contributions (*R*_HF_ and *R*_MF_) are in the same range for atmospheres without CO_2_. In contrast, in 5% O_2_ in CO_2_, both contributions were highly differentiated, with the *R*_MF_ being higher than the *R*_HF_. Mid frequencies are related to surface reactions and adsorption processes. In this case, the presence of CO_2_ could interfere with the oxygen adsorption, increasing the resistance for these processes.^48^ The activation energies (*E*_a_) for the polarization resistances with both principal contributions are compared in Table 1. The *E*_a_ for *R*_p_ for the CO_2_-free environments had a value similar to that of the *E*_a_ for the resistances at high and mid frequencies, indicating that both contributions had the same relevance. In addition, the *E*_a_ for *R*_p_ in CO_2_ was closer to *E*_a_ for the *R*_MF_, indicating that, in CO_2_ environments, the surface exchange reactions were limited by the adsorption processes. These results suggest that the material could potentially be used as an oxygen electrode in solid-oxide cells (SOC) in CO_2_-rich environments to electrochemically supply oxygen in oxycombustion processes.

**Table 1:** Activation Energy (*E*_a_) for the Global and Different Contributions and the Polarization Resistance at 850 °C

	***E***_**a**_ **(*R***_**p**_**)**	***E***_**a**_ **(*R***_**HF**_**)**	***E***_**a**_ **(*R***_**MF**_**)**	***R***_**p**_ **(850 °C)**
	**eV**	**eV**	**eV**	**Ω·cm**^**2**^
**21% O**_**2**_	1.23	1.08	1.36	0.353
**5% O**_**2**_	1.11	1.01	1.31	0.472
**5% O**_**2**_ **95% CO**_**2**_	1.34	0.93	1.63	0.488

### 3.3 Oxygen Permeation Studies

The dual-phase membrane consisting of 40% vol. CMO and 60% vol. CTO sintered at 1200 °C exhibited a remarkable increase in total conductivity compared to NFO/CTO. In order to enhance the permeation properties, a porous catalytic layer of the same material, 40CMO/60CTO, was added on both sides of the dual-phase membrane. Two *bulk* membranes with a thickness of ∼650 μm and catalytic layers with a thickness of ∼30 μm and an active surface of 64 mm^2^ were employed here.

**Figure 7:**
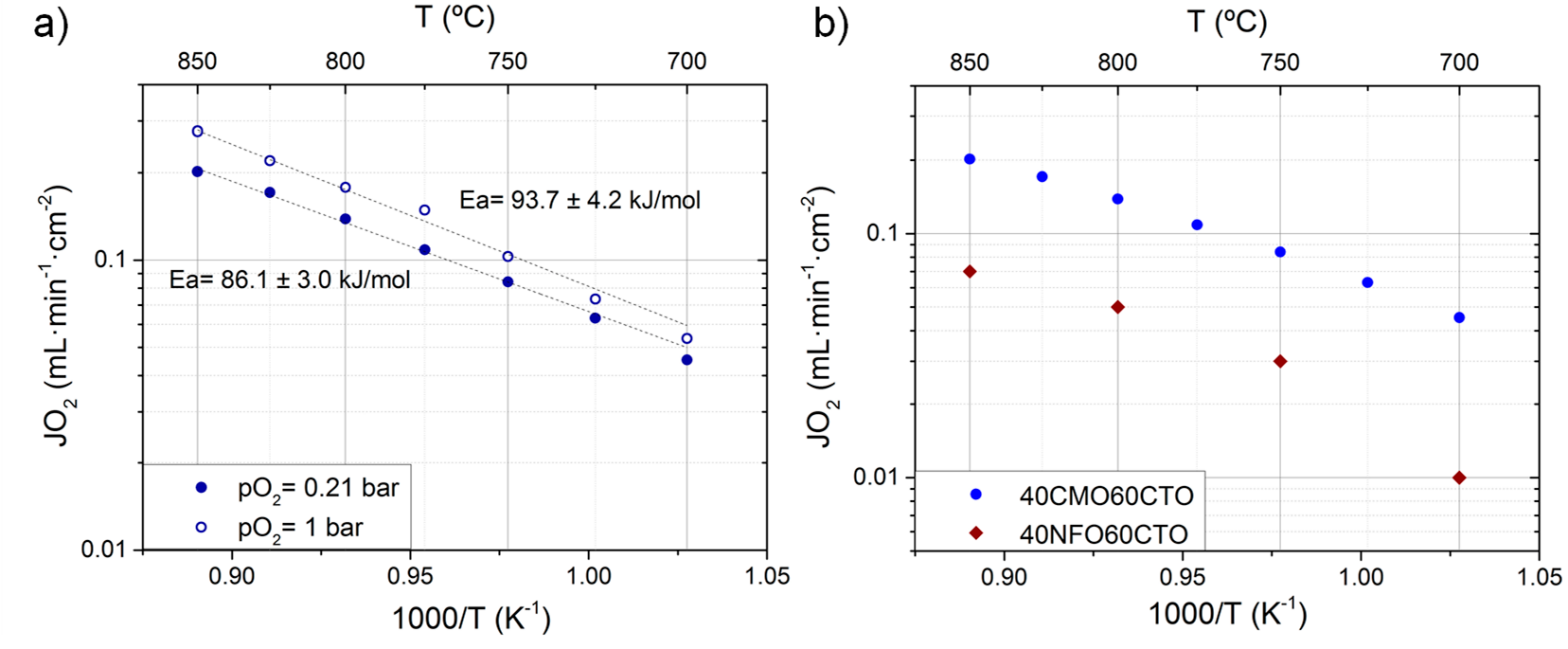
(a) Oxygen permeation of a 680 μm-thick CMO/CTO dual-phase membrane under air and pure O_2_ environment. (b) Oxygen permeation of CMO/CTO and NFO/CTO dual-phase membranes under air at different temperatures.^16^

The oxygen permeation experiments were performed in air on the feed side and Ar on the sweep side. The flows for those chambers were 100 mL·min^–1^ of air in the feed chamber and 150 mL·min^–1^ for the sweep chamber, analyzing the oxygen permeation from 850 to 700 °C. Furthermore, it was measured with pure oxygen at the feed side, equivalent to pressurized air conditions (air at 5 bar). Oxygen permeation results for both conditions are represented in Figure 7 and Table S5.

Figure 7a shows the oxygen permeation flux under air and pure oxygen for the CMO/CTO membrane. At 850 °C, the CMO/CTO membrane permeates 0.21 mL·min^–1^·cm^–2^, and when fed with pure oxygen, the permeation reached a value of 0.28 mL·min^–1^·cm^–2^ at 850 °C. This permeation is higher than that achieved for the same ratio composition for NFO/CTO membrane (Figure 7b) that produced 0.07 mL·min^–1^·cm^–2^ at 850 °C elsewhere.^16^ Furthermore, the performance is similar to literature reports by Yi et al.^34^ using a dual-phase membrane consisting of CMO and CGO, with values of 0.17 mL·min^–1^·cm^–2^ at 850 °C. The activation energies for both feeds were very similar, 86.1 and 93.7 kJ·mol^–1^ for air/Ar and O_2_/Ar, respectively. These activation energy values are lower than those achieved for the NFO/CTO membrane in air/Ar in the range 800–700 °C, which was more than 110 kJ·mol^–1^ for all compositions,^16^ suggesting that gas exchange is improved in the CMO/CTO membranes.

In addition, with the oxygen permeation values, it is possible to calculate the ambipolar conductivity of these dual-phase membranes, as shown in eq 7,
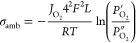
7where σ_amb_ is the ambipolar conductivity, *L* is the thickness of the membrane, *J*_O_2__ is the oxygen permeation for the dual-phase membrane, *T* is the temperature of operation, and *P*_O_2__^′^ and *P*_O_2__^″^ are the oxygen partial pressure in the sweep and the feed side, respectively. With these data and the total conductivity of the dual-phase material and the electronic conductivity of the electronic phase, it is possible to calculate the ionic conductivity of the CTO (eq 8)
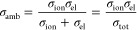
8where σ_tot_ is the total conductivity and σ_el_ is the total conductivity for the electronic phase. Table 2 depicts the ambipolar conductivity and the ionic conductivity values determined using eqs 7 and 8, respectively, for the NFO/CTO and CMO/CTO membranes at 800 °C under an air/Ar atmosphere.

**Table 2:** Oxygen Permeation and Conductivities for Dual-Phase Materials at 800 °C under Air/Ar Atmospheres

	***J***_**O_2_**_**(mL·min**^**–1**^**·cm**^**–2**^**)**	***σ***_**tot**_**(S·cm**^**–1**^**)**	***σ***_**amb**_**(S·cm**^**–1**^**)**	***σ***_**ion**_**(S·cm**^**–1**^**)**
**40NFO/60CTO**	0.05	0.07	0.11	0.03
**40CMO/60CTO**	0.14	6.31	0.29	0.03

**Figure 8:**
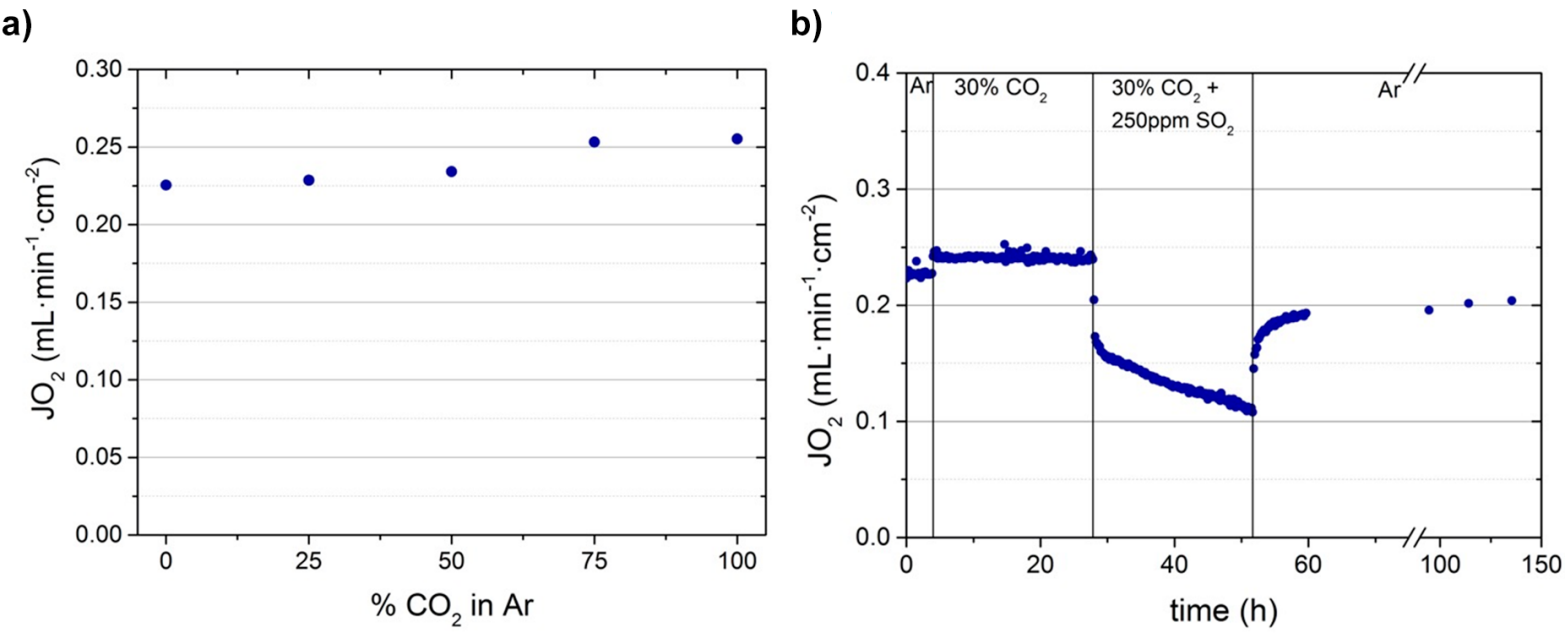
Oxygen permeation (stability studies) of a 680 μm-thick CMO/CTO membrane with 100 mL·min^–1^ of air at the feed side and 150 mL·min^–1^ of the different mixes at the sweep side at 850 °C: (a) different concentrations of CO_2_ (0–100%) and (b) 24 h of 30% CO_2_ and 24 h of 30% CO_2_ and 225 ppm of SO_2_ environments.

For both studies, the ionic conductivity calculated was 0.030 S·cm^–1^, similar to the total conductivity values of CTO under these conditions (0.034 S·cm^–1^), confirming that the fluorite phase is the only phase responsible for ionic conductivity in the membrane. One of the main advantages of dual-phase membranes is their stability in acidic gas environments, such as in the presence of CO_2_ or SO_2_. Two different experiments were performed to evaluate the performance of these membranes under these atmospheres. Figure 8a shows the oxygen permeation in different concentrations of CO_2_ (0–100%) in the sweep side, and Figure 8b shows the oxygen permeation and stability test for 150 h in Ar, 30% CO_2_ in Ar, and 30% CO_2_ + 250 ppm of SO_2_ in Ar. In Figure 8a, the total flows of the feed side and sweep side are 100 mL·min^–1^ of air in the feed chamber and 150 mL·min^–1^ of the mixture (CO_2_ + Ar) in the sweep chamber at 850 °C.

Here, the oxygen permeation increased with increasing CO_2_ concentrations on the sweep side, from 0.22 to 0.25 mL·min^–1^·cm^–2^, at 850 °C. This behavior was also described for NFO/CTO dual-phase membranes^16^ and ascribed to the better sweep and thermal-transfer properties of CO_2_ with respect to Ar.^52^ The maximum oxygen permeation was achieved with 100% CO_2_ in the sweep side. This is a sign of the absence of any undesired effect or reaction between the CO_2_ and both crystalline phases. This enhancement in oxygen permeation under CO_2_ at 850 °C is also shown in the experiment depicted in Figure 8b. However, in the presence of SO_2_, the permeation of the O_2_ decreased progressively for 24 h, evidencing that SO_2_ poisoned the membrane, blocking the active sites. Significantly, when CO_2_ and SO_2_ were removed from the stream, the level of the O_2_ permeation increased again until 0.20 mL·min^–1^·cm^–2^. However, the initial oxygen permeation level achieved at the stability test of 0.22 mL·min^–1^·cm^–2^ was not recovered, indicative of nonreversible membrane evolution on the SO_2_ stream.

When comparing with the NiFe_2_O_4_/Ce_0.8_Tb_0.2_O_2−δ_ (NFO/CTO) membrane,^16^ the oxygen permeation for the CMO/CTO membrane shows around 2 times higher values than for the NFO/CTO membrane at 850 °C in air/argon, viz., 0.22 and 0.11 mL·min^–1^·cm^–2^, respectively. This oxygen permeation increase was maintained under a CO_2_ environment, with 0.24 and 0.13 mL·min^–1^·cm^–2^ for CMO/CTO and NFO/CFO membranes, respectively. However, under SO_2_ environments, the loss of permeation capacity was more accentuated for the CMO/CTO membrane than for the NFO/CTO membrane. This suggests membrane degradation in the presence of SO_2_, which will be analyzed in the next section.

In this study, the CMO/CTO dual-phase membrane permeation in air at 850 °C was 0.21 mL·min^–1^·cm^–2^. This permeation value is very close to the standard values in dual-phase membranes at these temperatures with this thickness (around 600 μm) (Table 3).^5^ Furthermore, under CO_2_ environments, this CMO/CTO exhibits the highest permeation flux with 0.25 mL·min^–1^·cm^–2^ with pure CO_2_ in the sweep chamber. In addition, the oxygen permeation of this membrane in such environments is very stable over time. These results illustrate that CMO/CTO composites are promising candidates for OTMs in oxycombustion processes operating in high CO_2_ environments, especially if compared with state-of-the-art materials;^5^ see Table 3.

**Table 3:** Oxygen Permeation Results from Dual-Phase MIEC Membranes at 850 °C with Inert Gas (He and Ar) and CO_2_^a^

**Materials**	***L*****(μm)**	***J***_**O**_**2**__**(mL·min**^**–1**^**·cm**^**–2**^**)**	***T*****(°C)**	**Atm.*****p*****O**_**2**_^**feed**^**/*p*O**_**2**_^**sweep**^	**Ref.**
**Ce**_**0.9**_**Gd**_**0.1**_**O**_**2−δ**_**–Ba**_**0.5**_**Sr**_**0.5**_**Co**_**0.8**_**Fe**_**0.2**_**O**_**3−δ**_	500	0.8	875	air/He	(28)
	500	0.2	875	air/CO_2_	
**Ce**_**0.8**_**Nd**_**0.2**_**O**_**2−δ**_**–Nd**_**0.5**_**Sr**_**0.5**_**Fe**_**0.8**_**Al**_**0.2**_**O**_**3−δ**_	600	0.25	850	air/He	(53)
	600	0.1	850	air/CO_2_	
**Ce**_**0.9**_**Pr**_**0.1**_**O**_**2−δ**_**–Pr**_**0.6**_**Sr**_**0.4**_**Fe**_**0.99**_**Bi**_**0.01**_**O**_**3−δ**_	600	0.15	850	air/He	(29)
	600	0.05	850	air/CO_2_	
**Ce**_**0.8**_**Tb**_**0.2**_**O**_**2−δ**_**–NiFe**_**2**_**O**_**4**_ **+Pr**_**6**_**O**_**11**_	680	0.13	850	air/Ar	(33)
	680	0.13	850	air/CO_2_	
**Ce**_**0.8**_**Tb**_**0.2**_**O**_**2−δ**_**–Co**_**2**_**MnO**_**4**_	680	0.22	850	air/Ar	this study
	680	0.25	850	air/CO_2_	

a All of the membranes have a thickness (*L*) between 500 and 700 μm. Extracted from ref (5).

### 3.4 Post-Mortem Structural Characterization

As seen in Figure 8b, the oxygen flux of the CMO/CTO membrane dropped progressively with time on the SO_2_ (225 ppm) stream. Even so, when the SO_2_ is removed, the permeation flux increases again but does not recover the initial flux (Figure 8). In order to assess possible structural degradations, the side of the membrane exposed to the SO_2_ environment was analyzed by XRD (Figure S4). Here, the membranes were compared before the permeation test and after exposure to SO_2_ during the oxygen permeation test. From the XRD patterns, neither structural changes nor the presence of secondary phases or impurities could be ascertained.

**Figure 9:**
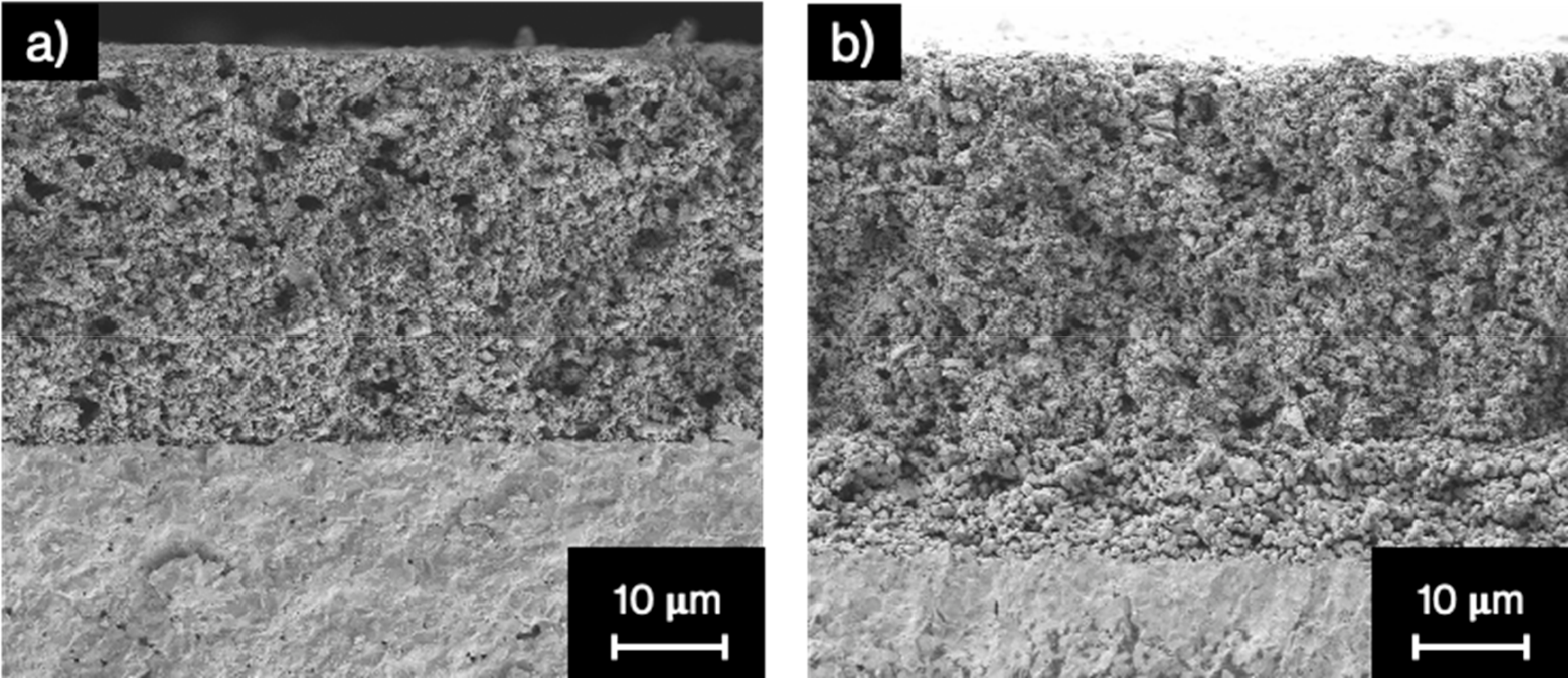
Cross-section image from SEM of the CMO/CTO membrane after CO_2_ and SO_2_ stability test: (a) catalytic layer from the feed side (air); (b) catalytic layer from the sweep side (CO_2_ and SO_2_).

Figure 9 shows SEM cross-sectional images of both sides of the membrane after the SO_2_ treatment, revealing microstructural evolution. As expected, the catalytic layer and the membrane on the feed side (air) were in good condition, Figure 9a, with a thickness of around 30–25 μm. However, microstructural changes can be inferred on the sweep side, as shown in Figure 9b. The catalytic layer seems unaltered, but a new porous layer appeared in the top part of the membrane, with a thickness of almost 10 μm. This porosity was previously seen by Garcia-Fayos et al. on the NFO/CTO membrane after exposure to SO_2_.^16^ One possible explanation for this morphological change is the reaction between SO_2_ and doped ceria. Several studies have revealed that the CeO_2_ forms Ce_2_(SO_4_)_3_^54,55^ in the presence of SO_2_ and O_2_. This poisoning increases with the oxygen content, which could explain the constant decrease in oxygen permeation in the presence of SO_2_ and O_2_. The formation of Ce_2_(SO_4_)_3_ could block the active sites (oxygen vacancies), decreasing the permeation with the increase of Ce_2_(SO_4_)_3_ into the doped ceria. The porosity on the membrane will be formed in the transition between Ce_2_(SO_4_)_3_ and CeO_2_ after the exposition to SO_2_. However, Ce_2_(SO_4_)_3_ could not be detected with the XRD equipment used in this study. The degraded zone was also analyzed with EDS, and sulfur could not be detected, as shown in Figure S5. Thus, although the formation of cerium sulfate seems the most probable reason for the membrane degradation, we could not corroborate its presence with the physicochemical characterization performed here.

## 4 Conclusions

The use of Co_2_MnO_4_ spinel as the electronic phase in the composite membranes was assessed. Here, we proved that the higher electronic conductivity of CMO with respect to the well-studied spinel (NiFe_2_O_4_, NFO) enables one to boost the oxygen transport in these dual-phase membranes. Namely, the increase in the total conductivity for the dual-phase membrane (CMO/CTO) leads to higher oxygen-permeation flux values with respect to NFO/CTO membranes, e.g., 0.22 and 0.11 mL·min^–1^·cm^–2^ at 850 °C under air/Ar, respectively. Furthermore, the CMO/CTO membrane exhibits the highest permeation in CO_2_ environments compared to the literature and excellent stability during a prolonged time, 0.24 and 0.13 mL·min^–1^·cm^–2^ for CMO/CTO and NFO/CFO membranes, respectively. This oxygen permeation is the highest for this membrane (>600 μm-thick) obtained in CO_2_ environments.^5^ The SO_2_-driven membrane poisoning was more evident than in previous studies for similar dual-phase membranes due to its increase in oxygen permeation compared to that study.^16^ In addition, similar surface-morphology evolution on the membrane was detected, but any presence of sulfur elements was inferred in the post-mortem characterization. This study illustrates that CMO/CTO composites could be promising candidates for OTMs or oxygen electrodes in CO_2_-capture-enabling oxycombustion processes.

## Supporting Information Available

SEM analysis of membrane cross sections, including a sample exposed to SO_2_; electrochemical impedance spectra and fitted parameters of impedance spectra as a function of temperature and *p*O_2_; oxygen permeation flux, including experimental error values; and XRD analysis of fresh and spent membranes (PDF)
