# Supplementary material for: Co2MnO4/Ce0.8Tb0.2O2−δ Dual-Phase Membrane Material with High CO2 Stability and Enhanced Oxygen Transport for Oxycombustion Processes
Source: ACS Appl Energy Mater. 2023 Dec 18;7(1):302–11. doi: 10.1021/acsaem.3c02606 (PMC10777685; doi:10.1021/acsaem.3c02606)
Supplement: Supplementary file 1 — ae3c02606_si_001.pdf [file ae3c02606_si_001.pdf]

Supporting Information

$Co_2MnO_4/Ce_{0.8}Tb_{0.2}O_{2-\delta}$ dual-phase membrane material with high CO_2 stability and enhanced oxygen transport for oxycombustion processes

Marwan Laqdiem[1], Julio Garcia-Fayos[1], Alfonso J. Carrillo[1], Laura Almar[1], María Balaguer[1], María Fabuel[1], José M. Serra*[1]

[1] Instituto de Tecnología Química (Universitat Politècnica de València – Consejo Superior de Investigaciones Científicas), av. Los Naranjos s/n, E-46022 Valencia, Spain

** Corresponding author. Tel: + 34.963879448 E-mail: jmserra@itq.upv.es*

Table S1. Fitted parameters of the equivalent circuit for CMO/CTO catalytic layer at 850 ºC in different pO_2.

Fit	Units	0.21	0.16	0.11	0.05
R_p	$\Omega \cdot cm^2$	0.35 ± 0.02	0.38 ± 0.02	0.41 ± 0.02	0.47 ± 0.02
R_{HF}	$\Omega \cdot cm^2$	0.181 ± 0.008	0.177 ± 0.008	0.208 ± 0.008	0.228 ± 0.007
Ceq_{HF}	F/cm^2	$4.86 \cdot 10^{-5}$	$4.81 \cdot 10^{-5}$	$5.14 \cdot 10^{-5}$	$5.34 \cdot 10^{-5}$
ω_{HF}	Hz	$1.81 \cdot 10^{4}$	$1.87 \cdot 10^{4}$	$1.49 \cdot 10^{4}$	$1.31 \cdot 10^{4}$
R_{MF}	$\Omega \cdot cm^2$	0.172 ± 0.009	0.200 ± 0.008	0.18 ± 0.01	0.202 ± 0.009
Ceq_{MF}	F/cm^2	$7.33 \cdot 10^{-4}$	$7.11 \cdot 10^{-4}$	$1.10 \cdot 10^{-3}$	$1.33 \cdot 10^{-3}$
ω_{MF}	Hz	$1.26 \cdot 10^{3}$	$1.12 \cdot 10^{3}$	$8.08 \cdot 10^{2}$	$5.90 \cdot 10^{2}$
R_{LF}	$\Omega \cdot cm^2$			0.019 ± 0.003	0.042 ± 0.004
Ceq_{LF}	F/cm^2			0.76	0.48
ω_{LF}	Hz			11.13	7.85

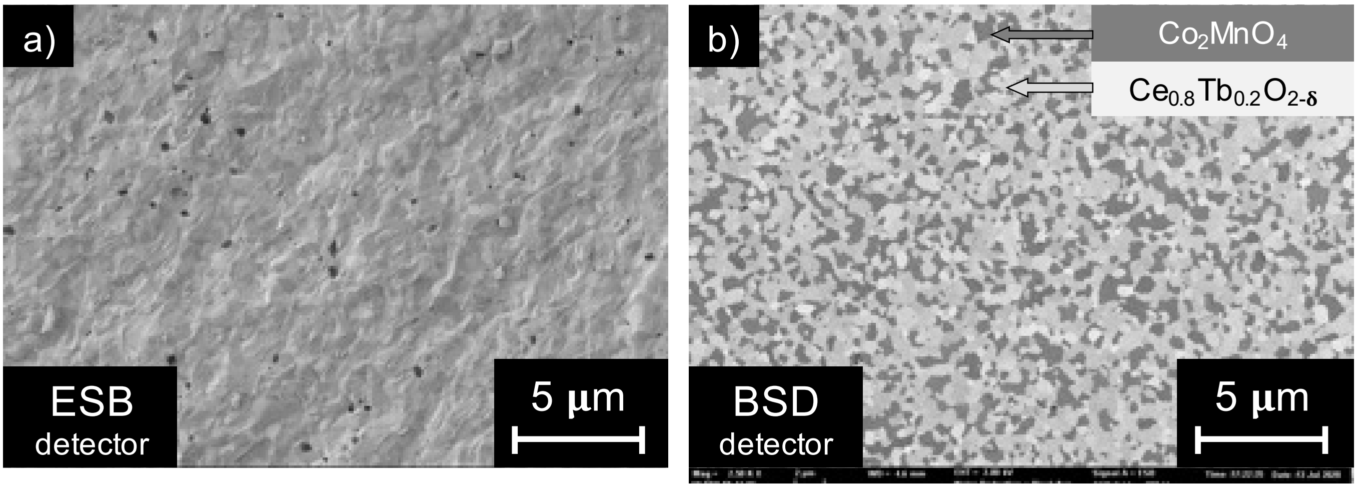

Figure S1. Cross-section for the bulk-section of a CMO/CTO dense membrane a) SEM cross-section and b) ESB images. Labels for the different phases CMO and CTO phases are indicated.

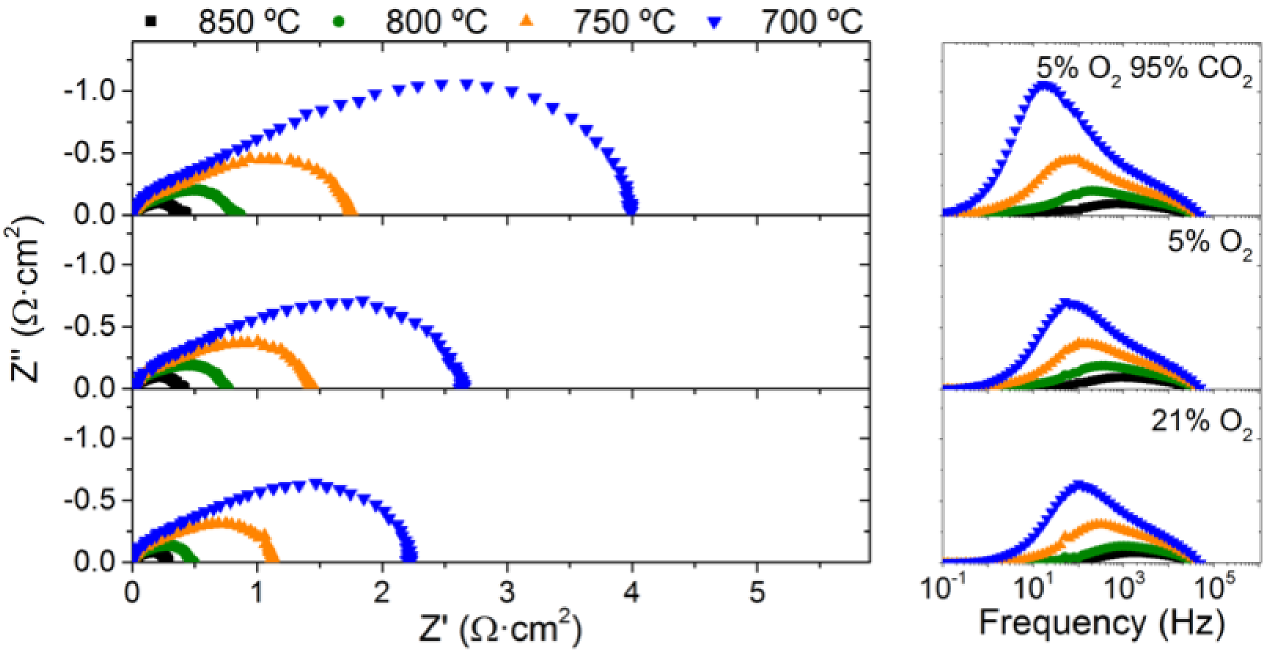

Figure S2. Polarization resistance and different fitted resistances associated with the HF, MF, and LF contributions of the electrode 40CMO/60CTO at different temperatures and different environments: air, at 5% of O_2 in Ar and 5% of O_2 and 95% of CO_2.

Table S2. Fitted parameters of the equivalent circuit for CMO/CTO catalytic layer at different temperatures in air.

Fit	Units	850ºC	800ºC	750ºC	700ºC
R_p	$\Omega \cdot cm^2$	0.35 ± 0.02	0.52 ± 0.03	1.21 ± 0.1	2.36 ± 0.2
R_{HF}	$\Omega \cdot cm^2$	0.181 ± 0.008	0.24± 0.01	0.47 ± 0.06	0.98 ± 0.12
Ceq_{HF}	F/cm^2	$4.85 \cdot 10^{-5}$	$6.16 \cdot 10^{-5}$	$6.04 \cdot 10^{-5}$	$8.90 \cdot 10^{-5}$
ω_{HF}	Hz	$1.81 \cdot 10^4$	$1.08 \cdot 10^4$	$5.58 \cdot 10^3$	$1.82 \cdot 10^3$
R_{MF}	$\Omega \cdot cm^2$	0.172 ± 0.009	0.28 ± 0.01	0.74 ± 0.05	1.38 ± 0.11
Ceq_{MF}	F/cm^2	$7.33 \cdot 10^{-4}$	$8.06 \cdot 10^{-4}$	$8.67 \cdot 10^{-4}$	$1.23 \cdot 10^{-3}$
ω_{MF}	Hz	$1.26 \cdot 10^3$	$6.94 \cdot 10^2$	$2.47 \cdot 10^2$	$9.36 \cdot 10$

Table S3. Fitted parameters of the equivalent circuit for CMO/CTO catalytic layer at different temperatures in 5% of O_2.

Fit	Units	850ºC	800ºC	750ºC	700ºC
R_p	$\Omega \cdot cm^2$	0.47 ± 0.02	0.85 ± 0.12	1.53 ± 0.14	2.78 ± 0.36
R_{HF}	$\Omega \cdot cm^2$	0.228 ± 0.006	0.43± 0.06	0.70 ± 0.08	1.16 ± 0.19
Ceq_{HF}	F/cm^2	$5.34 \cdot 10^{-5}$	$6.39 \cdot 10^{-5}$	$7.56 \cdot 10^{-5}$	$9.45 \cdot 10^{-5}$
ω_{HF}	Hz	$1.31 \cdot 10^{4}$	$5.89 \cdot 10^{3}$	$2.99 \cdot 10^{3}$	$1.45 \cdot 10^{3}$
R_{MF}	$\Omega \cdot cm^2$	0.202 ± 0.009	0.40 ± 0.05	0.83 ± 0.07	1.62 ± 0.17
Ceq_{MF}	F/cm^2	$1.33 \cdot 10^{-3}$	$1.53 \cdot 10^{-3}$	$1.61 \cdot 10^{-3}$	$1.59 \cdot 10^{-3}$
ω_{MF}	Hz	$5.90 \cdot 10^{2}$	$2.59 \cdot 10^{2}$	$1.19 \cdot 10^{2}$	$6.19 \cdot 10$
R_{LF}	$\Omega \cdot cm^2$	0.042 ± 0.004	0.016 ± 0.005		
Ceq_{LF}	F/cm^2	0.48	1.94		
ω_{LF}	Hz	7.85	5.19		

Table S4. Fitted parameters of the equivalent circuit for CMO/CTO catalytic layer at different temperatures in 5% of O_2 and 95% CO_2

Fit	Units	850ºC	800ºC	750ºC	700ºC
$\boldsymbol{R_p}$	$\Omega\cdot cm^2$	0.49 ± 0.02	0.88 ± 0.03	1.80 ± 0.02	4.15 ± 0.07
$\boldsymbol{R_{HF}}$	$\Omega\cdot cm^2$	0.160 ± 0.006	0.235± 0.008	0.40 ± 0.01	0.71 ± 0.03
$\boldsymbol{Ceq_{HF}}$	F/cm^2	$5.89\cdot10^{-5}$	$6.60\cdot10^{-5}$	$8.55\cdot10^{-5}$	$9.15\cdot10^{-5}$
$\boldsymbol{\omega_{HF}}$	Hz	$1.69\cdot10^{4}$	$1.03\cdot10^{4}$	$4.67\cdot10^{3}$	$2.45\cdot10^{3}$
$\boldsymbol{R_{MF}}$	$\Omega\cdot cm^2$	0.26 ± 0.01	0.61 ± 0.01	1.40 ± 0.01	3.44 ± 0.04
$\boldsymbol{Ceq_{MF}}$	F/cm^2	$2.00\cdot10^{-3}$	$1.60\cdot10^{-3}$	$1.25\cdot10^{-3}$	$1.05\cdot10^{-3}$
$\boldsymbol{\omega_{MF}}$	Hz	$5.94\cdot10^{2}$	$2.09\cdot10^{2}$	$7.09\cdot10$	$2.31\cdot10$
$\boldsymbol{R_{LF}}$	$\Omega\cdot cm^2$	0.071 ± 0.006	0.040 ± 0.007		
$\boldsymbol{Ceq_{LF}}$	F/cm^2	0.51	1.83		
$\boldsymbol{\omega_{LF}}$	Hz	4.35	2.20		

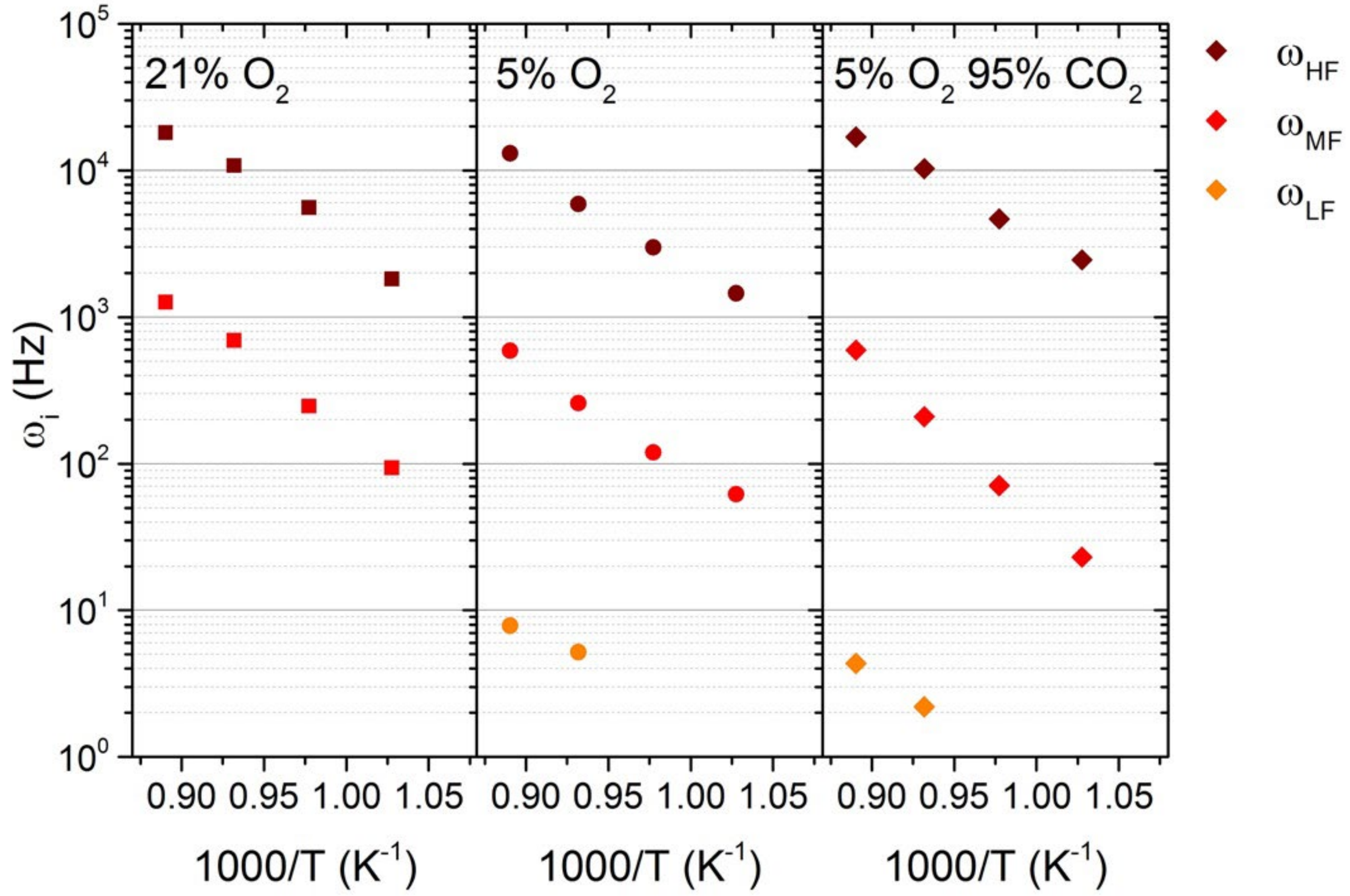

Figure S3. Different fitted maximum frequency for the different contributions (ω_{HF}, ω_{MF} and ω_{LF}) measured at different environments (air, 5% O_2 in Ar and 5% O_2 in CO_2) from 750 ºC to 850 ºC for symmetrical cells with 40CMO/60CTO electrodes.

Table S5. Oxygen permeation values for CMO/CTO dual-phase membrane under air and pure O_2 environment

Temperature (°C)	JO_2 ($mL \cdot min^{-1} \cdot cm^{-2}$)	
	oxygen	Air
850	0.2771 ± 0.0006	0.2020 ± 0.0009
825	0.220 ± 0.001	0.1712 ± 0.0006
800	0.1780 ± 0.0006	0.139 ± 0.001
775	0.149 ± 0.002	0.1086 ± 0.0006
750	0.103 ± 0.002	0.0842 ± 0.0006
725	0.073 ± 0.001	0.0631 ± 0.0005
700	0.054 ± 0.002	0.0453 ± 0.0004

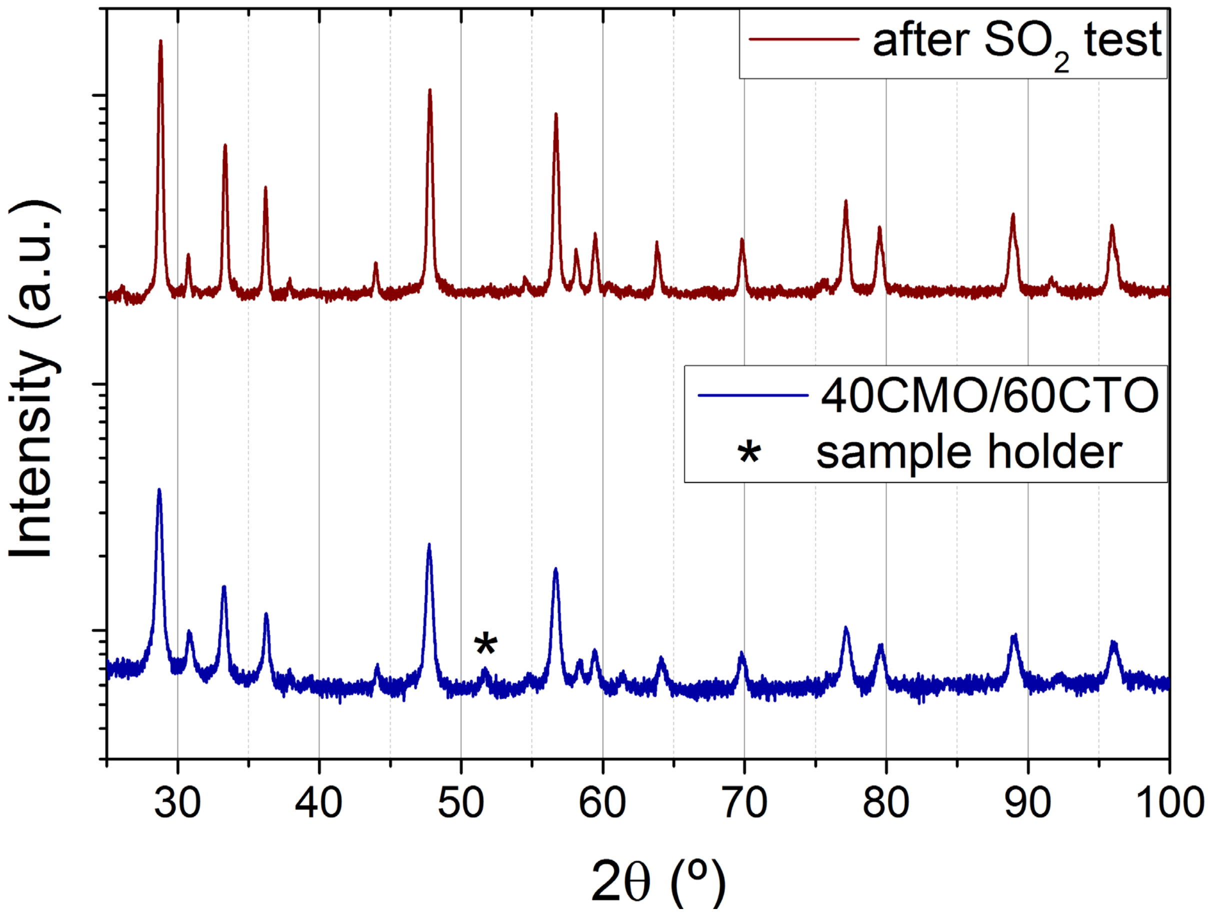

Figure S4. XRD for CMO/CTO before (bottom) and after the stability test performed with SO_2 (top).

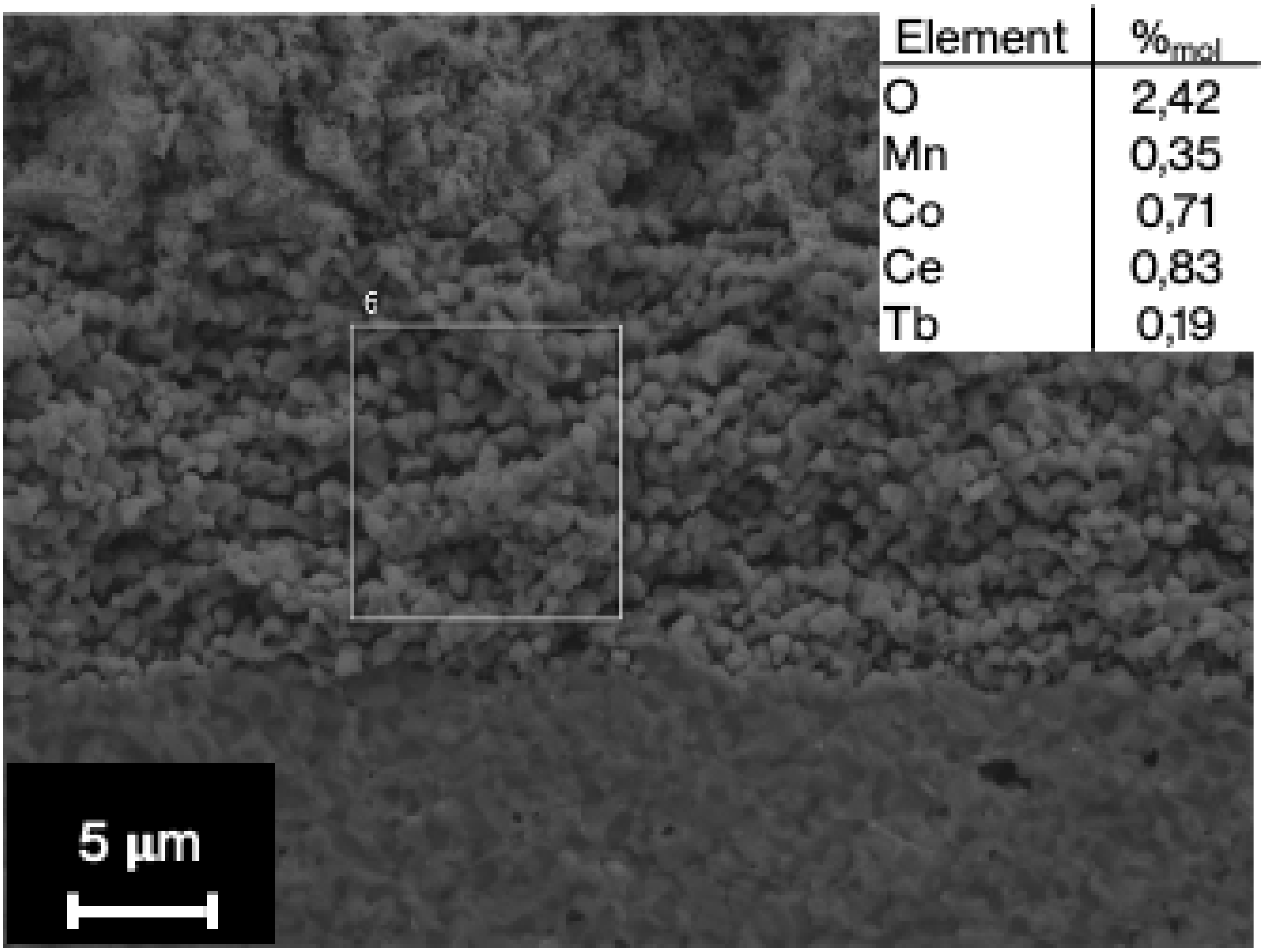

Figure S5. EDS SEM analysis for part of the degraded area after being exposed to SO_2 environments.
